# Native mixed microbe inoculants (M1H) optimize soil health to promote *Cajanus cajan* growth: the soil fungi are more sensitive than bacteria

**DOI:** 10.3389/fmicb.2025.1521064

**Published:** 2025-03-05

**Authors:** Zexun Liu, Chengcheng Luo, Kang Zheng, Yongtao Sun, Jie Ru, Yaner Ma, Xinru Zhang, Yong Zhou, Jiayao Zhuang

**Affiliations:** ^1^Collaborative Innovation Center of Sustainable Forestry in Southern China of Jiangsu Province, Nanjing Forestry University, Nanjing, China; ^2^East China Academy of Inventory and Planning of NFGA, Hangzhou, China; ^3^National Forestry and Grassland Bureau Forest and Grass Survey Planning Institute, Beijing, China; ^4^Beijing Liangshui River Administration Office, Beijing, China

**Keywords:** *Cajanus cajan*, growth promoting effect, soil nutrients, soil fungal and bacterial communities, FUNGuild

## Abstract

Microbial inoculant is widely used in plant growth and crop production. However, the effect of native mixed microbial inoculants on soil microbiota and plant growth remain to be elucidated. Here, we used pot experiment for 5 months to determine the microbial inoculants treatments with growth-promoting effect on *Cajanus cajan*, such as M1P (*Serratia marcescens*) treatment and M1H treatment: the mixture of M1P and M45N (*Paenibacillus polymyxa*), and investigate the effect of these inoculants on the capacity of soil nutrients and rhizosphere microbiomes in promoting *C. cajan* growth. Further, the adaptability of these strains to environmental stress (temperature and pH) was determined by using stress-resistant growth experiment. The results showed that M1H treatment resulted in soil nutrients consumption and led to substantial alterations in the microbial community that were more effective in promoting *C. cajan* growth. The enhanced plant growth observed with M1H inoculation may be due to its impact on the soil micro-environment, particularly through increasing beneficial genera (e.g., *Cunninghamella*, *Mortierella*, *Chryseolinea*, and *Bacillus*) and decreasing potential genera (e.g., *Zopfiella* and *Podospora*). In addition, at the genus level (top 10), the effect of M1H inoculation on soil fungal community was higher than that of bacteria, which shows that the change of soil fungal community after M1H inoculation was more sensitive than that of bacteria. Spearman correlation analysis further revealed that the abundance of *Cunninghamella*, *Mortierella*, *Chryseolinea*, *Zopfiella* and *Podospora* were the key factors affecting *C. cajan* growth. Moreover, FUNGuild function prediction clearly indicated distinct differences in the fungal functions of CK, MIP and M1H treatment, in which a lower relative abundance of saprotroph fungi in M1H treatment compared to CK, these results may confirmed the possibility of decreasing the abundance of *Zopfiella* and *Podospora* under M1H treatment. Taken together, our findings highlight the role of M1H inoculant in promoting *C. cajan* growth and ameliorating soil health, and providing valuable insight of using native mixed microbial inoculants to cultivate *C. cajan* and optimize soil micro-environment.

## 1 Introduction

*Cajanus cajan* is a perennial woody plant, which belongs to the subfamily Papilionidae of Leguminosa (Gnanesh et al., 2011). It is widely consumed as food due to its high nutritional value (Rao et al., 2002). In addition, *C. cajan* has a high tolerance to different stresses, such as barren resistance, salt tolerance, drought tolerance, and so on. Thus, it is regarded as a pioneer tree species for the prevention and control of soil erosion. These characteristics have increased the utilization rate of *C. cajan* worldwide (Lazcano et al., 2021). Due to the rapid decline of the available land and the sharp decline in soil fertility, it is necessary to improve both land utilization rates and plant growth rates, which is crucial given the current situation (Dastager et al., 2010). The application of chemical fertilizer is the most common method to promote plant growth and increase crop yield. The rapid development of agriculture has increased the dependency of agriculture on chemical fertilizers. Chemical fertilizers increase plant growth and crop yield in a short time but adversely affect the soil environment, resulting in abnormal levels of soil nutrients and a decreased abundance of beneficial microbes, and in turn, aggravating soil degradation and posing a potential threat to human health (Liu et al., 2023; Tian et al., 2021). Thus, microbial fertilizer containing beneficial microbes has become the hot spot of agricultural research studies as it is stable and eco-friendly.

Soil microbes are a vital part of the soil ecosystem (Baldock and Skjemstad, 2000; Wang et al., 2017). They play an important role in the soil nutrient cycle, such as the decomposition of organic matter and regulation of soil nutrient levels (Semchenko et al., 2022). Certain soil microbes improve soil quality and the soil micro-ecological environment by dissolving insoluble nutrients and secreting plant hormones. Besides, they also increase plant’s soil nutrient consumption and promote plant growth (Li et al., 2022; Tian et al., 2021).

Previous studies have shown that application of microbial inoculant could affect soil microbial diversity and community structure and directly or indirectly promote plant growth. For instance, *Trichoderma harzianum* and *Bacillus* inoculants increased the abundance of beneficial soil microbes and inhibited the growth of pathogenic bacteria resulting in increased tomato yield (Cheng et al., 2020, 2021). Chen et al. (2020) reported that beneficial microbial fertilizer effectively inhibited the accumulation of soil pathogens and ameliorated the rhizospheric microbial community structure, resulting in increased growth of *Chrysanthemum*. Zhuang et al. (2021) reported that *Penicillium*. NL-Z1 fungal inoculants induced and increased the abundance of *Mortierella*, increasing growth of *Acanthopanax* sp. Li et al. (2022) reported that the application of microbial fertilizer containing *Bacillus* inoculant increased the abundance of beneficial microbes, such as *Bacillus* (is included in the phylum *Firmicutes*) and *Actinomadura* (is included in the phylum *Actinobacteria*), and decreased the abundance of pathogenic fungi, such as *Fusarium* and *Phytophthora* in the rhizospheric soil of strawberry plants, increasing the yield of strawberries. Till now, numerous studies have investigated the effects of single microbial inoculants on plant growth and soil micro-ecological environment (El-Sharkawy et al., 2021). However, how native mixed microbial inoculants can help us to promote plant growth, understand the correlation and of soil nutrients, soil microbial community structure, and plant growth remain unclear.

Soil microbial community structure is an important biological index to evaluate plant growth (Mapelli et al., 2012; Tian et al., 2021; Zhu et al., 2014). The interspecific correlations among microbes, including induction, transformation, coexistence, and antagonism, play a significant role in shaping the structure and diversity of the soil microbial community, thereby affecting plant growth. Previous studies have shown that optimizing the soil microbial community structure could directly or indirectly determine the adaptability of plants to the environment (Kos et al., 2009; Samaddar et al., 2021; Stefan et al., 2021), especially the altered abundance of beneficial and pathogenic microbes in the soil, which could reflect the soil’s health. This also demonstrates that understanding the altered microbial community structure of plant growth is crucial. Thus, understanding the changes in microbial community structure will help us to unravel microbial mechanisms concerning plant growth. Although the well-studied nature of single microbial inoculants, there are few studies on the effects of inoculation with microbial inoculants from *C. cajan* rhizosphere, such as *Serratia marcescens* and *Paenibacillus polymyxa*, especially the effects of the mixed microbial inoculants of *S. marcescens* and *P. polymyxa* on the growth of *C. cajan* is unknown. Therefore, it is essential to study the external benefits of mixed microbial inoculants of *S. marcescens* and *P. polymyxa* and the internal relationships between microbes.

In this study, we conducted a pot experiment on *C. cajan* plant for 5 months using native microbial inoculants, i.e., *P. polymyxa* (M45N) and *S. marcescens* (M1P), which were isolated from the rhizospheric soil of *C. cajan*. We also used high-throughput Illumina MiSeq (ITS and 16S) sequencing technology to investigate taxonomic changes in the soil’s bacterial and fungal communities in response to different microbial inoculants. We hypothesized that (1) soil nutrients and microbial community structure in the rhizosphere would be affected by different microbial inoculants; (2) mixed microbial inoculants would have a stronger growth-promoting effect on *C. cajan* than single microbial inoculants; and (3) changes in the microbial community could be a potential factor affecting this ability. Consequently, these characteristics would further unveil the underlying mechanisms of microbial inoculants on *C. cajan* growth. In conjunction with the premise of the rational use of space resources, the specific objectives of this study were (1) to assess the effect of different microbial inoculants on growth indexes (plant height, plant ground diameter and dry weight), soil nutrients, soil microbial diversity, microbial community structure, and function, and (2) to evaluate the environmental factors influence the fungal and bacterial communities composition. This study assessed and confirmed the feasibility and effectiveness of native mixed microbial inoculants in promoting plant growth, providing a theoretical basis and sustainable management for the cultivation and development of *C. cajan*.

## 2 Materials and methods

### 2.1 The source, isolation and screening of rhizosphere microbes, and preparation of microbial inoculants

*Cajanus cajan* seeds were purchased from a flower seed market in Jiujiang City, Jiangxi province, China, and were planted in the Baguazhou experimental field in Qixia District, Nanjing in mid-May 2022. At the end of November, the soil rhizospheric samples from healthy *C. cajan* were collected in the experimental field. The soil samples were stored in a 4°C ice box and brought back to the soil and water conservation laboratory of Nanjing Forestry University for microbial screening experiments.

The microbial screening experiments step as follows: A 10-fold serial dilution of soil samples was smeared on NA (nutrient agar, for cultivating bacteria: 3 g of beef extract, 10 g of peptone, 5 g of NaCl, 20 g of agar, and 1,000 mL of deionized water, pH 7.0–7.2) and PDA (potato dextrose agar, for cultivating fungi: 6 g of potato flour, 20 g of glucose, 18 g of agar, and 1,000 mL of deionized water, pH 7.0–7.2) to isolate bacteria and fungi, respectively. The agar plates were incubated at 28°C for 3 days. Morphologically distinct colonies were subjected to purification following subculturing. Seven purified strains were obtained, namely M1N, M2N, M3N, M45N, M1P, M2P, and M45P. Among these, M1N, M2N, M3N, M45N, M1P, and M2P were bacterial isolates (NA) and M45P was a fungal isolates (PDA) (Supplementary Figure S1). Purified strains were prepared into microbial inoculants with the values of 0.8–1.2 under OD600 (equivalent to 0.8–1.2 × 10^8^ cfu/mL), and 60 mL of them was applied to the rhizosphere soil for the pot experiment of *C. cajan*.

### 2.2 Pot experiment

The experiment was conducted in a greenhouse at Baguazhou, Nanjing Province, China. The temperature of the greenhouse was maintained at 30 ± 5°C, a relative humidity of 65%, and with adequate sunlight exposure. The initial farmland soil was air-dried, screened by a 5 mm mesh, and mixed with vermiculite and perlite in a ratio of 3:1:1. The mixed soils were filled with 2.5 kg per pot (30 cm in diameter, 25 cm in depth). The initial soil properties are determined in Supplementary Table S1.

On April 29, 2023, *C. cajan* seeds were pretreated prior to the pot experiment. Firstly, we soaked *C. cajan* seeds in distilled water for 12 h, filtered the water, and then soaked them in a 0.5% sodium hypochlorite solution for 1 min to disinfect the seeds (Solorzano and Malvick, 2011). Later, the disinfected seeds were washed with pure water and later kept in the seedling cup to germinate for 2 week. The seeds germinated into seedlings by May 15, and the seedlings with similar height growth (about 6-8 cm in height) were transplanted into pots with 2.5 kg soil. One healthy seedling was planted in each pot. Nine treatments with five replicates were designed, thus a total of 45 pots were designed in this study. The main treatments were as follows: (1) CK: contained sterile LB culture solution (without strain derivatives); (2) M1P: *S. marcescens* inoculant; (3) M1H: inoculants of the mixture contained M45N (*P. polymyxa*) and M1P; (4) Other treatments, including M1N, M2N, M3N, M2P, M45N, and M45P treatments, had no growth-promoting effect on *C. cajan* growth (Supplementary Figure S2). Thus, only the treatments with significant differences compared to CK, such as M1P and M1H treatments, were analyzed in the subsequent fungal and bacterial communities analysis.

One week after the seedlings were transplanted (on May 22), microbial inoculants with an OD600 of 0.8–1.2 were prepared, and 60 mL of them was applied to the rhizosphere soil in each treatment. The first day of microbial inoculants application were recorded as day one of the experiment, and inoculation was repeated every other month (on May 22, June 22, July 22, August 22, and September 22). Samples were taken after 5 months, from May 22 to October 22, 2019. During the whole experimental period, potted plants were randomly placed in the greenhouse and rearranged every other month. Identical agronomic management measures were then implemented across all treatments for the study.

### 2.3 Stress-resistant growth experiment

The stress-resistant growth experiment was conducted in the Soil and Water Conservation Laboratory of Nanjing Forestry University and included temperature and pH stress resistance tests. For the temperature stress resistance test, derivatives of M1P (1-mm-diameter disk), M45N (1-mm-diameter disk), and M1H (0.5-mm diameter disk of M1P and M45N, respectively) purified through multiple generations were inoculated into 250 mL conical flasks containing 100 mL of LB culture medium. The LB medium was prepared with 10 g of peptone, 5 g of yeast extract, 5 g of NaCl, and 1,000 mL of deionized water, adjusted to pH 7.2. The cultures were shaken evenly and incubated at temperature gradients of 10, 20, 30, 40, 50, and 60°C for 3 days, with three replicates per temperature condition. Strain concentrations in the culture solutions were measured every 12 h using an ultraviolet–visible spectrophotometer at OD600, and these values represented the strain concentrations. For the pH stress resistance test, sulfuric acid and sodium hydroxide were used to adjust the pH of solutions to values of 5, 6, 7, 8, 9, and 10. LB culture media were prepared by replacing deionized water with these pH-adjusted solutions. Derivatives of M1P, M45N, and M1H were inoculated into 250 mL conical flasks containing 100 mL of the pH-adjusted LB culture medium. The cultures were incubated at 35°C ± 0.2°C for 3 days, with three replicates for each pH condition. Strain concentrations were measured every 12 h using an ultraviolet–visible spectrophotometer at OD600. The experiment was designed with two primary factors (temperature and pH), six secondary factors (six temperature gradients and six pH gradients), three biological replicates, and three strains, resulting in a total of 108 conical flasks. This setup ensured a comprehensive assessment of microbial stress resistance under varying environmental conditions.

The strains of M1P and M45N were ascertained based on microbial screening experiments (Supplementary Figure S3) and pot experiments (Supplementary Figures S2, S4). Besides, the strain of M45N and M1P were identified based on morphological characteristics and 16S rDNA analysis, which were identified *P. polymyxa* and *S. marcescens*, respectively. The preparation of microbial inoculants step as follows: To prepare inoculants of M45N, M1P, and their mixture (M1H), purified isolates were inoculated on NA plates, respectively, until their derivatives covered half of the plate. Derivatives were inoculated into a 250 mL Erlenmeyer flask containing 100 mL LB (Luria Bertani) medium. Cultures were incubated at 25°C and 200 rpm for 48 h. After 48 h, the cultivating bacteria of the suspension was measured (UV-8000T, Shanghai Metash Instruments Co., Ltd.) at OD600 using a UV–visible spectrophotometer. The values of OD600 in the bacterial solutions was ensured to be in the range of 0.8–1.2 by dilution or continuous culturing. Microbial inoculants were prepared by diluting the culture 100 times. These microbial inoculants were used in *C. cajan* pot experiment.

### 2.4 Plant and soil sampling

The pot experiment was concluded after 5 months, and the samples of plants and rhizospheric soil in the pot were collected for indexes measurement. For plant, vernier calipers and tape were utilized to measure the plant ground diameter and plant height of the seedlings, as the growth indexes of *C. cajan*. Plant height and plant ground diameter were measured before each harvest. The dry weight of plant was determined after drying at 60°C for 24 h until constant weight in oven. For soil, rhizospheric soils adhered to the roots of plants from the range of 0–20 cm were collected by shaking method (Gently swing by hand for 2 min, with a frequency of about 60 times/min), and the fine roots in rhizosphere soils were completely removed with tweezers to reduce the influence on the experimental determination. Thus, a total of 15 rhizospheric soil samples were collected, and each of which was about 100 g. The soil samples were stored in the self-sealing bags. Each soil sample was divided into two parts and brought back to the laboratory on ice. One part of the sample was stored at a − 80°C for soil microbial community analysis, and the other part of the sample was air-dried to determine for the chemical properties analyses of the soil.

### 2.5 Soil chemical properties analyses

The levels of total nitrogen (TN), total potassium (TK), hydrolyzed nitrogen (AN), and available potassium (AK) were determined by using the Semi-micro Kjeldahl method (De Falco et al., 2004; J et al., 1984), sodium hydroxide melting flame photometry, alkaline hydrolysis diffusion method, and flame photometry with NH_4_OAc extraction, respectively. The level of soil organic carbon (OC) was determined using external heating with concentrated sulfuric acid and potassium dichromate. Besides, a 10 g soil sample into a 50 mL beaker, add distilled water with no CO_2_ according to the water-soil ratio of 2.5:1, stir it with a stirrer for 1 min, and let it stand for 30 min, then determine it with a pH meter.

### 2.6 Soil DNA extraction, PCR amplification, and high-throughput gene sequencing

A total of 0.25 g of rhizosphere soil from each potted soil samples were used for DNA extraction. The complete genomic DNA of potted soil samples was extracted using the e.z n.a.^®^ Soil DNA Kit (Omega Bio-Tek, Norcross, GA, United States) and detailed steps were referred to the built-in instructions. The concentration of extracted DNA was detected by Nanodrop RND-2000 (NanoDrop Technologies, Wilmington, DE, United States). After extraction, the extracted genomic DNA was detected on using 1% agarose gel electrophoresis. Then, two universal primers 338F (5’-ACTCCTACGGGAGCAGCAG-3′) - 806R (5’-GGACTACHGGGTWTCTAAT-3′) (Ma et al., 2020) and ITS1F (5′-GATGAAGA ACGYAGYRAA-3′) - ITS2R (5’-GCTGCGTTCT TCAT CATGATGC-3′) (Adams et al., 2013) were used to perform PCR amplification and MiSeq sequencing on the V3–V4 region of the bacterial 16SrRNA gene and the fungal ITS1 region. The PCR reaction consisted of 12.5 μL 2 × Premix Taq™ (TaKaRa. Bio Inc. Shiga, Japan), 1 μL of each primer (10 μm), 2 μL of DNA extract (5–20 ng), and 9 μL of dd H_2_O to a final volume of 25 μL. The PCR amplification conditions as follows: denaturated at 95°C for 3 min, followed by 35 cycles of denaturation at 95°C for 30 s, annealing at 55°C for 30 s, annealing at 55°C for 30 s, and a final extension step at 72°C for 10 min. The PCR products of the same samples were mixed and detected by using 2% agarose gel electrophoresis and purified by using an AxyPrepDNA gel recovery kit (Axygen Biosciences, U.S.). Referring to the preliminary quantitative results of electrophoresis, the PCR products were quantified using QuantiFluor^TM^-ST blue fluorescence quantitative system (Promega company). The DNA samples were quantified and mixed according to the sequencing quantity requirements of each sample. The DNA samples were sequenced using Mothur (V.1.36.1). The columns were filtered to remove the chimera to obtain the optimized sequence. The workflow as follows: FASTP v. 0.19.6 was used to perform quality control on the original sequences to remove low-quality reads, and FLASH v. 1.2.11 was used for splicing to obtain longer sequences (Magoč and Salzberg, 2011; Callahan et al., 2016). UPARSE v. 11 was used for operational taxonomic unit (OTU) clustering after quality control splicing, and the chimeras were removed according to 97% similarity to acquire the optimized DNA sequences, which were divided into operational classification units (OTUs) (Edgar et al., 2011). MiSeq sequences of purified amplicons were high-throughput sequenced by Guangzhou Jidi’ao Technology Service Co., Ltd. (Guangzhou, China) using Illumina^®^ MiSeq sequencer (Illumina, San Diego, CA, United States).

### 2.7 Bioinformatics analysis and statistical analysis

Statistical analysis of the soil’s chemical properties and growth indexes of *C. cajan* was carried out by applying one-way analysis of variance (ANOVA) and the new multiple range method to the data. All analyses were conducted with SPSS statistical software package, version 20.0 (IBM, United States). The significance for statistical tests was accepted at *p* < 0.05.

Microbial (fungal and bacterial) diversity indexes including the Chao1 and Shannon indexes were calculated using the “vegan” R package^1^. R’s default ggplot2 (v3.5) package was used to make species composition analysis graphs and the vegan package for environmental correlation analysis to detect the relationship between environmental factors, samples, and microbial community. Besides, Welch’s *t*-test was used to compare differences in the relative abundance of the microbial phylum and genus level between treatments. The correlations between the environmental factors, samples, and microbial community were evaluated via redundancy analysis (RDA) using Canoco software (version 4.5). Prior to conducting the RDA analysis, we standardized the units of physicochemical parameters using R’s default vegan package to ensured uniformity and consistency in the data. Spearson’s correlation coefficient was employed to correlate the physical and chemical properties of soil and microbial communities in soil. Moreover, the “Vegan” package was used for principal coordinates analysis (PCoA) to determine the beta diversity of microbial communities based on Bray–Curtis dissimilarity, and the first two axes were then plotted using R package “ggplot2 (v3.5). For fungi (ITS gene data), based on their putative life history following ecological guild assignment sensu FUNGuild, the functional categories were assigned with the confidence of highly probable and probable (Nguyen et al., 2016). FUNGuild is a database for the comparison of fungal functions and linking fungal gene sequencing information with the ecological functions of fungi, as well as identifying the nutrient types used by fungi at the genus level and conducting the specific functional classifications. It was also used to study the fungal ecological functions in ecosystems (Tanunchai et al., 2023). For bacteria (16S gene data), the FAPROTAX v. 1.1 (functional annotation of transgenic taxa) database was used to perform rapid functional screening and grouping of 16S bacterial data from terrestrial ecosystems (Sansupa et al., 2021). Based on the OTU classification table of bacterial 16S, the FAPROTAX database was used to predict the potential biogeochemical cycling process of soil samples for functional annotation (Louca et al., 2016). All biological information analysis was carried out using the dynamic real-time interactive online data analysis platform^2^.

The isolates of M45N and M1P were deposited in the China Center for Type Culture Collection with the deposit numbers are M20221840 and M20221841, respectively. Additionally, the obtained sequences were uploaded to the NCBI database, and the registration numbers are OR976060 and OR976061, respectively.

## 3 Results

### 3.1 Growth of *C. cajan*

Compared with the control (CK), M1P and M1H treatments significantly increased the plant growth of *C. cajan*, including plant height, plant ground diameter, and dry weight, while M45N treatment had no significant differences (Table 1). Among these, M1H treatment as the optimal treatment, significantly increased the plant height, plant ground diameter, and dry weight by 35.58, 23.74, and 26.41% (*p* < 0.05), respectively. Specifically, the aboveground dry weight increased by 28.68%, while the underground dry weight increased by 21.81%, all showing statistically significant differences compared to CK (*p* < 0.05). Besides, M1P treatment exhibited similar effects on plant growth as M1H treatment in plant growth, and the growth-increasing effect of M1H treatment was greater than that of M1P treatment. Additionally, refer to Supplementary Figure S1 for the other treatments that had no growth-promoting effect on *C. cajan* growth.

**Table 1:** Effect of application of microbial inoculants on the growth of *Cajanus cajan*.

Indexes	CK	M45N	M1P	M1H
Height/cm	87.30 ± 4.36a	92.57 ± 5.86ab	108.17 ± 4.06b	118.37 ± 2.11c
Plant ground diameter/mm	6.15 ± 0.25a	6.80 ± 0.31ab	7.14 ± 0.20bc	7.61 ± 0.22c
Dry weight/g	654.72 ± 8.34a	684.56 ± 6.38ab	756.32 ± 5.42b	827.65 ± 7.33c
Aboveground-dry weight/g	438.66 ± 6.12a	449.07 ± 5.08a	513.71 ± 3.22b	564.46 ± 5.13c
Underground-dry weight/g	216.06 ± 4.22a	235.49 ± 5.20b	242.61 ± 5.20c	263.19 ± 6.20d

Different lowercase letters indicate the significant differences (*p* < 0.05).

### 3.2 Changes in soil properties under different microbial inoculants

**Figure 1:**
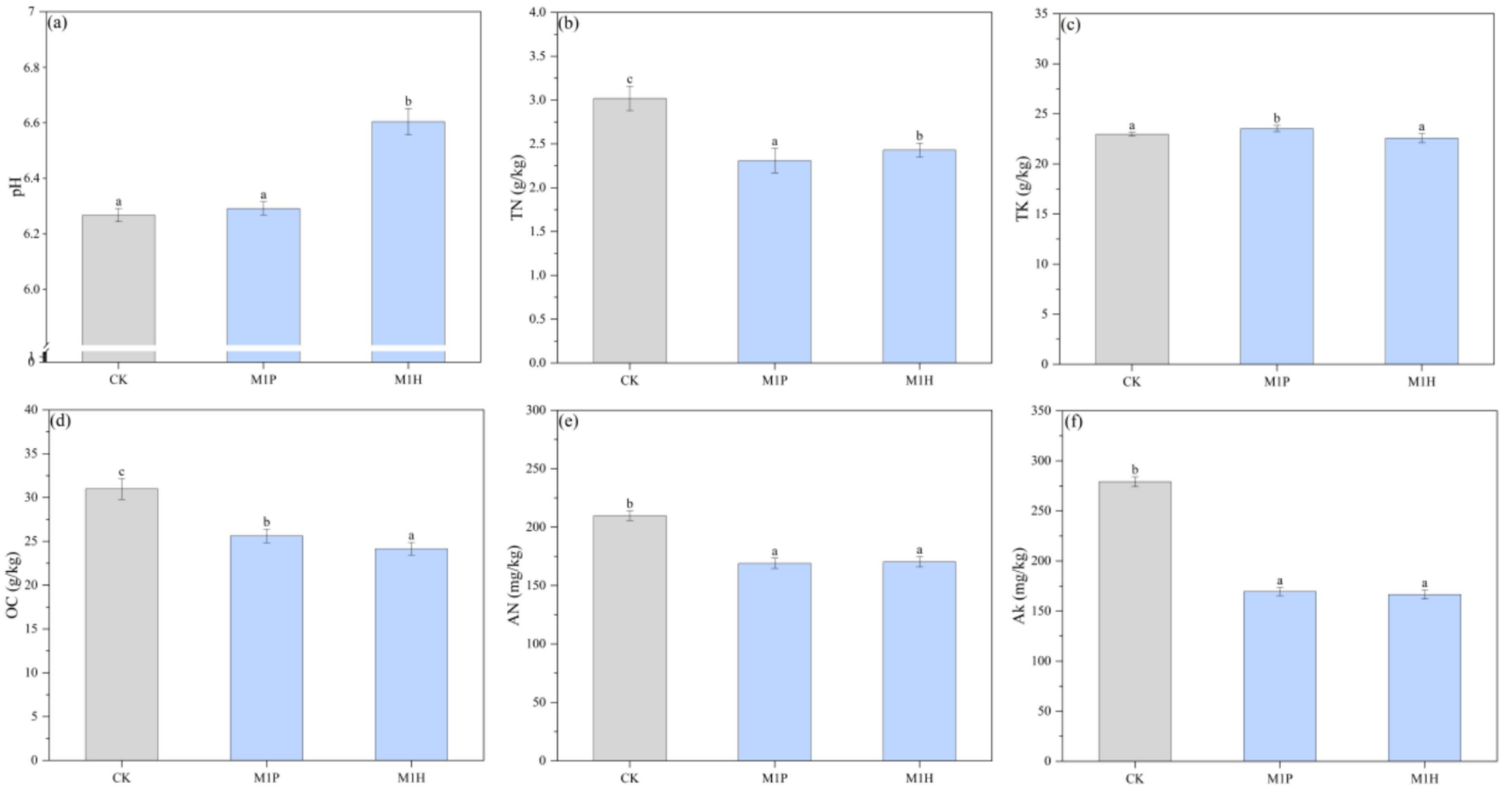
Variation in soil properties (soil pH **(A)**, TN **(B)**, TK **(C)**, OC **(D)**, AN **(E)**, and AK **(F)**) under different microbial inoculants. Here, OC, TN, AN, TK, and AK represent organic matter, total nitrogen, available nitrogen, total potassium, and available potassium, respectively. Different lowercase letters indicate the significant differences (*p* < 0.05).

As shown in Figure 1, compared with CK, M1P and M1H treatments alleviated soil acidification to a certain extent, and their pH values were 6.31 and 6.61, respectively, and significantly decreased the contents of OC, TN, AN, and AK in the soil (*p* < 0.05). Specifically, M1P and M1H treatments significantly decreased the OC content by 17.14 and 21.89% (*p* < 0.05), respectively, compared to CK, and significantly decreased the TN content by 26.13 and 19.23%, respectively. Moreover, decreasing the TN content did not increase the AN content, and significantly decreased it by 19.08 and 19.71% (*p* < 0.05) under M1P and M1H treatments, respectively. Besides, the contents of TK and AK showed a similar trend to that of TN and AN under M1P and M1H treatments.

### 3.3 Analysis of soil fungal and bacterial diversity

**Figure 2:**
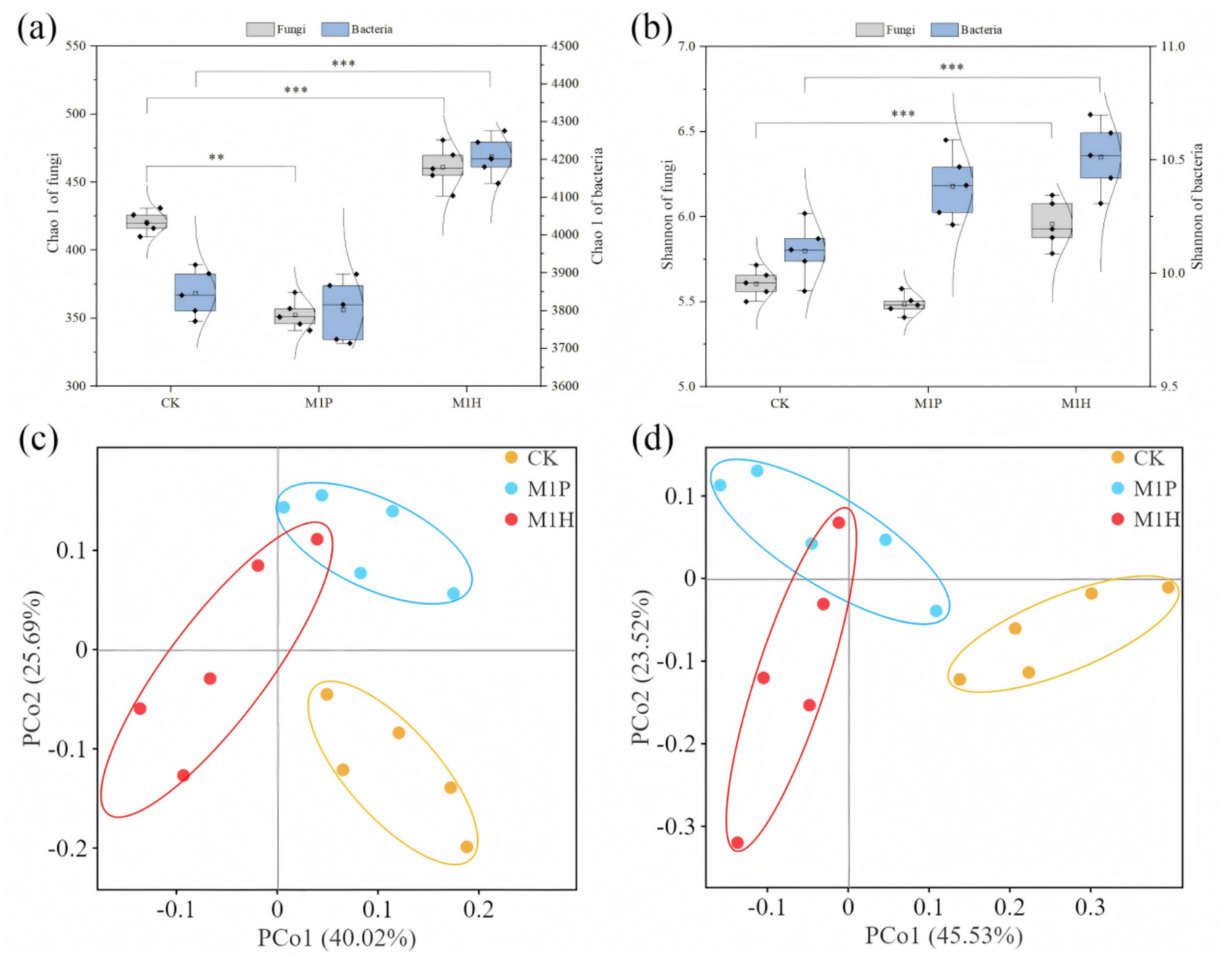
Variation in the alpha diversity **(A,B)** and principal coordinate analysis (PCoA) **(C,D)** of soil fungi **(A,C)** and bacteria **(B,D)** under different microbial inoculants. Here, *, ** and *** denote a significant difference between the different treatments at *p* < 0.05, *p* < 0.01 and *p* < 0.001, respectively.

The fungal and bacterial communities of 15 rhizospheric soil samples were analyzed using high-throughput Illumina MiSeq sequencing technology. A total of 1,662,391 fungal and 1,122,625 bacterial sequences were obtained from all samples, which were clustered into 6,176 fungal OTUs and 59,291 bacterial OTUs, and with an average sequencing coverage rate of 98% (Supplementary Figure S5). The OTUs dilution curve of the samples tended to be flat, indicating high-quality sequencing data were reasonable and reliable. The alpha diversity of fungal and bacterial communities in the rhizosphere soil samples was assessed using the Chao1 and Shannon indexes. Among then, Chao1 index represent the richness and uniformity of OTUs, and Shannon index represent the diversity and uniformity of microbial community (Figures 2A,B). The results showed that M1H treatment had significant effect on the alpha diversity of soil fungi and bacteria (*p* < 0.05), while M1P treatment had no significant effect, compared to CK. Specifically, Chao 1 index of soil fungi and bacteria were significantly increased by 10.34 and 9.41% under M1H treatment (*p* < 0.05), respectively, and by 6.61 and 2.67% to Shannon index, respectively (Figures 2A,B). In contrast, M1P treatment decreased the Chao 1 and Shannon indexes of soil fungi, and decreased Shannon index of soil bacteria, compared to CK. The findings reveal the influence of the fungal and bacterial alpha diversity changes caused by M1P and M1H treatments on *C. cajan* (see discussion section for details). The PCoA showed that the five replicates in each treatment in the fungal and bacterial communities were clustered together, indicating good repeatability between samples, and M1P and M1H treatments result in obviously deviated in the composition of the soil fungal and bacterial communities (Figures 2C,D). Specifically, the contribution rates of PCo1 and PCo2 to the differences in fungal species composition between treatments were 40.02 and 25.69%, and 45.53 and 23.52% to bacteria species composition, respectively.

### 3.4 Distribution of microbial community structures

**Figure 3:**
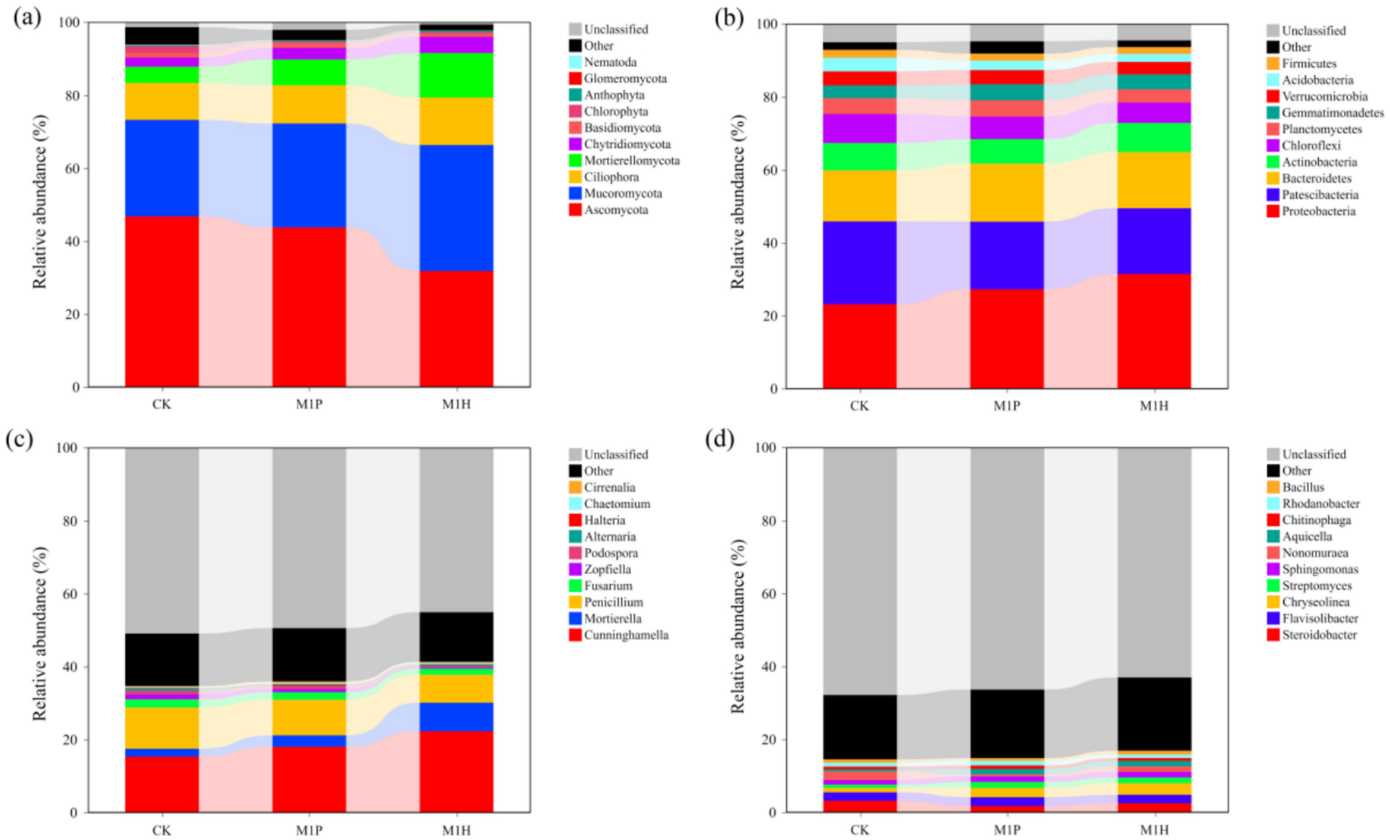
Relative abundance of fungal **(A,C)** and bacterial **(B,D)** communities at phylum **(A,B)** and genus **(B,C)** levels under different microbial inoculants.

**Figure 4:**
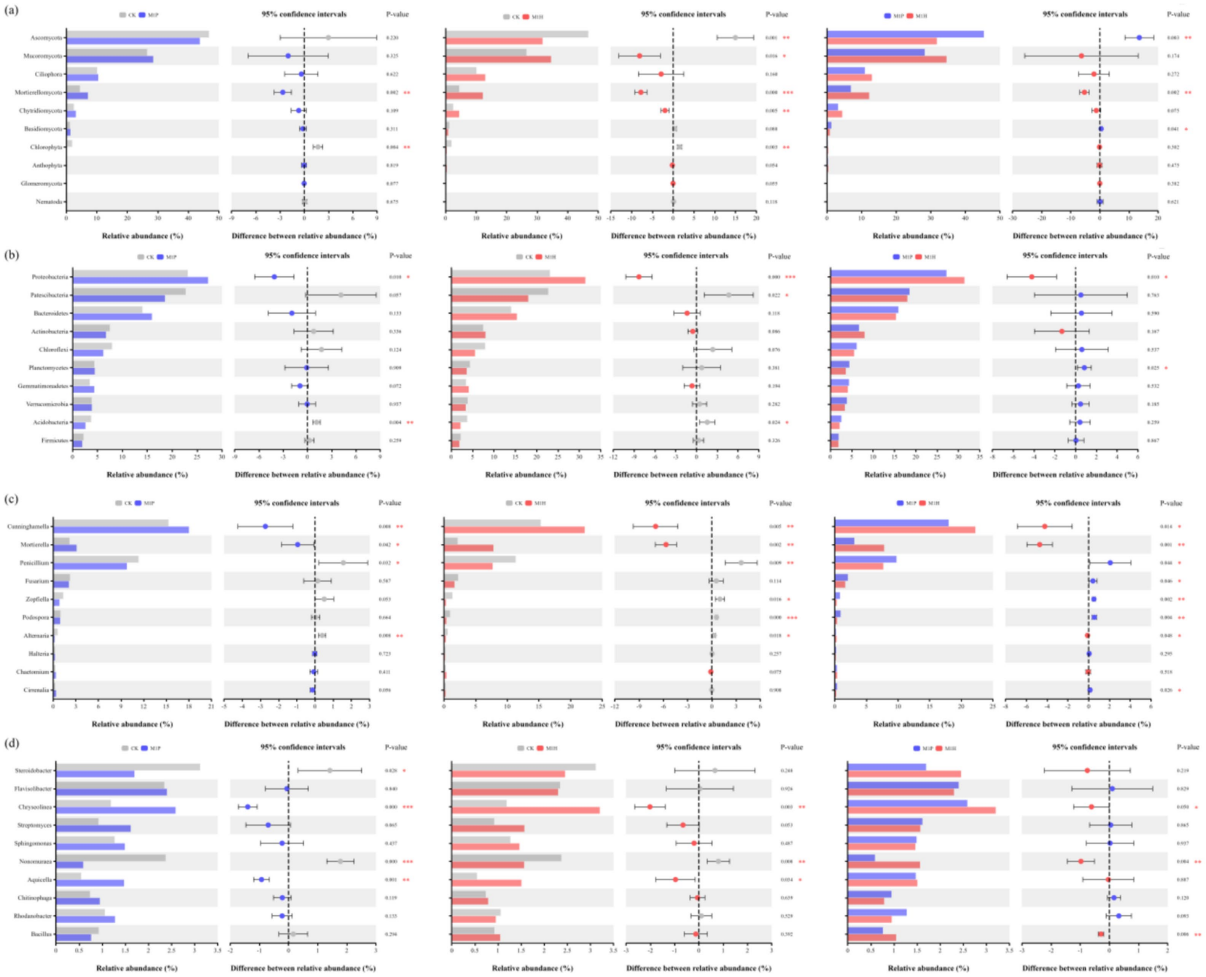
Student’s *t*-test bar plot on fungal **(A,C)** and bacterial **(B,D)** communities at phylum **(A,B)** and genus **(B,C)** levels with 95% confidence intervals. Here, *, ** and *** denote a significant difference between the two treatments at *p* < 0.05, *p* < 0.01 and *p* < 0.001, respectively. At the phylum and genus level, all phyla and genera with effective sequence abundances within the top 10 were screened for analysis. The abscissa is the species, and the ordinate is the relative abundance percentage and confidence intervals.

The shifts in fungal and bacterial (phylum and genus) communities compositions across all the treatments were investigated based on internal transcribed spacer region (ITS1) gene and the hyper-variable V3–V4 region (16S rDNA) of the 16S rRNA gene sequencing. Figures 3A,C demonstrate the fungal community at the phylum and genus levels in the soil samples. Of these phyla, *Ascomycota, Mucoromycota, Ciliophora, Mortierellomycota*, *Chytridiomycota* and *Basidiomycota* were the dominant phyla in fungal communities (relative abundance of these species exceeded 1% in all treatments). Compared to CK, the relative abundance of *Ascomycota* and *Chlorophyta* were significantly decreased by 32.00% (*p* = 0.001) and 79.79% (*p* = 0.003) under M1H treatment, respectively, while significantly increased the relative abundance of *Mucoromycota* (*p* = 0.016) and *Mortierellomycota* (*p* < 0.001) by 30.47 and 173.99%, respectively. Importantly, *Mucoromycota* (34.59%) replaced *Ascomycota* (31.80%) as the most abundant phylum under M1H treatment, which increased from 26.51 to 34.59%. M1P treatment had no significantly effected to the relative abundance of *Ascomycota*, *Mucoromycota* and *Chytridiomycota*. Besides, M1P and M1H treatments significantly increased the relative abundance of *Mortierellomycota* by 59.41% (*p* = 0.002) and 173.99% (*p* < 0.001), respectively. Meanwhile, as is shown in Figure 4A, the relative abundance of *Ascomycota* and *Mortierellomycota* had significantly different between M1P and M1H treatments. The relative abundance of *Ascomycota* were 31.80 and 43.78% (*p* = 0.003), respectively, and *Mortierellomycota* were 7.11 and 12.22% (*p* = 0.002), respectively, under M1P and M1H treatments. Other phyla were present at low levels. In addition, we further analyzed the changes of the top 10 species of fungal communities at the genus level (Figure 3C). Among then, *Cunninghamella*, *Mortierella*, *Fusarium*, *Penicillium*, *Zopfiella*, and *Podospora* were the top six fungal species in the genus level, which were the dominant genera in the fungal communities across all soil samples, and these species mainly belong to *Mucoromycota* (*Cunninghamella*), *Mortierellomycota* (*Mortierella*) and *Ascomycota* (*Fusarium*, *Penicillium*, *Zopfiella*, and *Podospora*). Compared to CK, M1P and M1H treatments significantly increased the relative abundance of *Cunninghamella* by 17.83% (*p* = 0.008) and 45.44% (*p* = 0.005), respectively, the relative abundance of *Mortierella* by 44.16% (*p* = 0.042) and 262.69% (*p* = 0.002), respectively. Besides, the relative abundance of *Penicillium* was significantly decreased by 13.77% (*p* = 0.032) and 32.12% (*p* = 0.009), respectively. Meanwhile, as is shown in Figure 4C, the relative abundance of *Cunninghamella*, *Mortierella*, *Penicillium*, *Zopfiella*, and *Podospora* had significantly different between M1P and M1H treatments. Compared to M1P treatment, M1H treatment significantly increased the relative abundance of *Cunninghamella* and *Mortierella* by 23.43% (*p* = 0.014) and 151.59% (*p* = 0.001), respectively, while significantly decreased relative abundance of *Penicillium*, *Zopfiella* and *Podospora* by 21.28% (*p* = 0.044), 60.39% (*p* = 0.002) and 58.86% (*p* = 0.004), respectively.

For bacteria, Figures 3B, 4B demonstrate the bacterial community at the phylum and genus levels in the soil samples. Of these phyla, *Proteobacteria*, *Patescibacteria*, *Bacteroidetes*, *Actinobacteria*, *Chloroflexi*, *Planctomycetes*, *Gemmatimonadetes*, *Verrucomicrobia*, *Acidobacteria*, and *Firmicutes* were considered dominant (contributed an average of 1% or more to total community composition) and were differentiated in all treatments. Compared to CK, M1P treatment significantly affected the relative abundance of *Proteobacteria* and *Acidobacteria*, and M1P treatment significantly affected the relative abundance of *Proteobacteria*, *Patescibacteria*, and *Acidobacteria.* Besides, *Proteobacteria* was the most dominant bacterial phyla in all the treatments. Compared to CK, M1P and M1H treatments significantly increased the relative abundance of *Proteobacteria* by 17.68% (*p* = 0.010) and 35.88% (*p* < 0.001), respectively. M1H treatment significantly increased the relative abundance of *Proteobacteria* by 15.49% (*p* = 0.010) compared with M1P treatment. Moreover, compared to CK, M1P and M1H treatments decreased the relative abundance of *Patescibacteria* by 18.29 and 20.54% (*p* = 0.022), respectively. In addition, we further analyzed the changes of bacterial community at the genus level (Figures 3D, 4D). Compared to CK, M1P and M1H treatments significantly affected the relative abundance of the relative abundance of *Steroidobacter*, *Chryseolinea*, *Nonomuraea*, and *Aquicella.* Compared to CK, M1P and M1H treatments significantly increased the relative abundance of *Chryseolinea* by 118.07% (*p* < 0.001) and 169.81% (*p* = 0.003), respectively, and M1H treatment increased the relative abundance of *Bacillu*s by 13.51%. Differently, compared to M1P treatment, M1H treatment significantly increased the relative abundance of *Chryseolinea*, *Nonomuraea* and *Bacillu*s by 13.73% (*p* = 0.050), 165.66% (*p* = 0.004) and 37.69% (*p* = 0.006), respectively.

### 3.5 Interaction between microbial communities and environmental variables in rhizosphere soil

**Figure 5:**
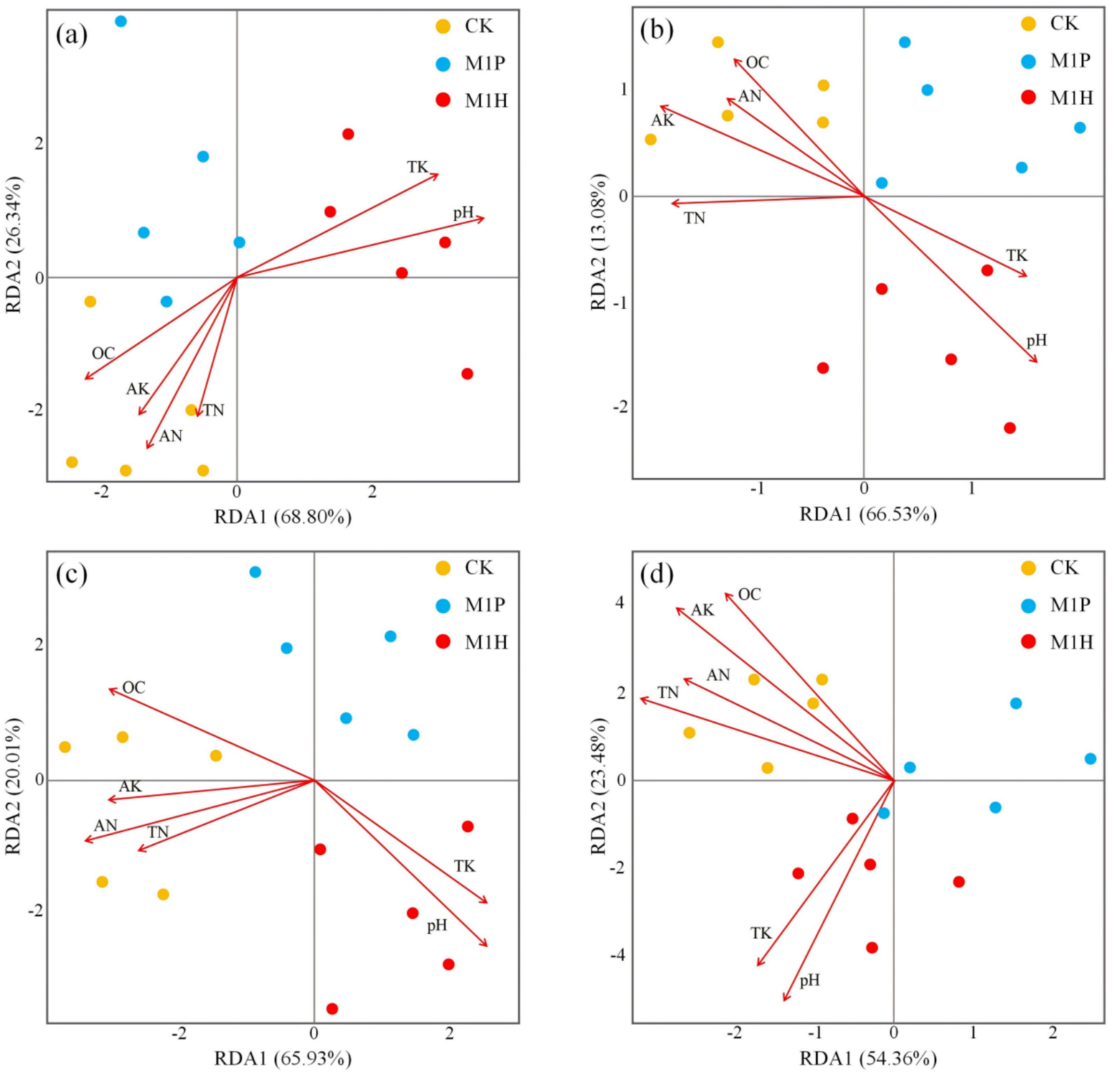
Redundancy Analysis (RDA) of the soil nutrients, soil samples, and phylum **(A,B)** and genus **(C,D)** of soil fungal **(A,C)** and bacterial **(B,D)** communities. Here, OC, TN, AN, TK, and AK represent organic matter, total nitrogen, available nitrogen, total potassium, and available potassium, respectively.

We analyzed the influence of soil properties on microbial community structure. As per the outcomes of RDA (Figure 5), the RDA of the soil fungal and bacterial communities explained the total variance of 95.14% (Figure 5A) and 79.61% (Figure 5B) at the phylum levels, respectively. Besides, the RDA of the soil fungal and bacterial communities explained the total variance of 85.94% (Figure 5C) and 76.84% (Figure 5D) at the genus levels, respectively. These indicated the reliability of the RDA, and microbial inoculants addition resulted in apparent changes in the soil microbial community structure. In addition, the fungal and bacterial community in CK treatment was positively correlated with OC, TN, AN, and AK, and negatively correlated with pH and TK (Figure 5). In contrast, the fungal and bacterial community in M1H treatment was negatively correlated with OC, TN, AN, and AK, and positively correlated with pH and TK (Figure 5).

**Figure 6:**
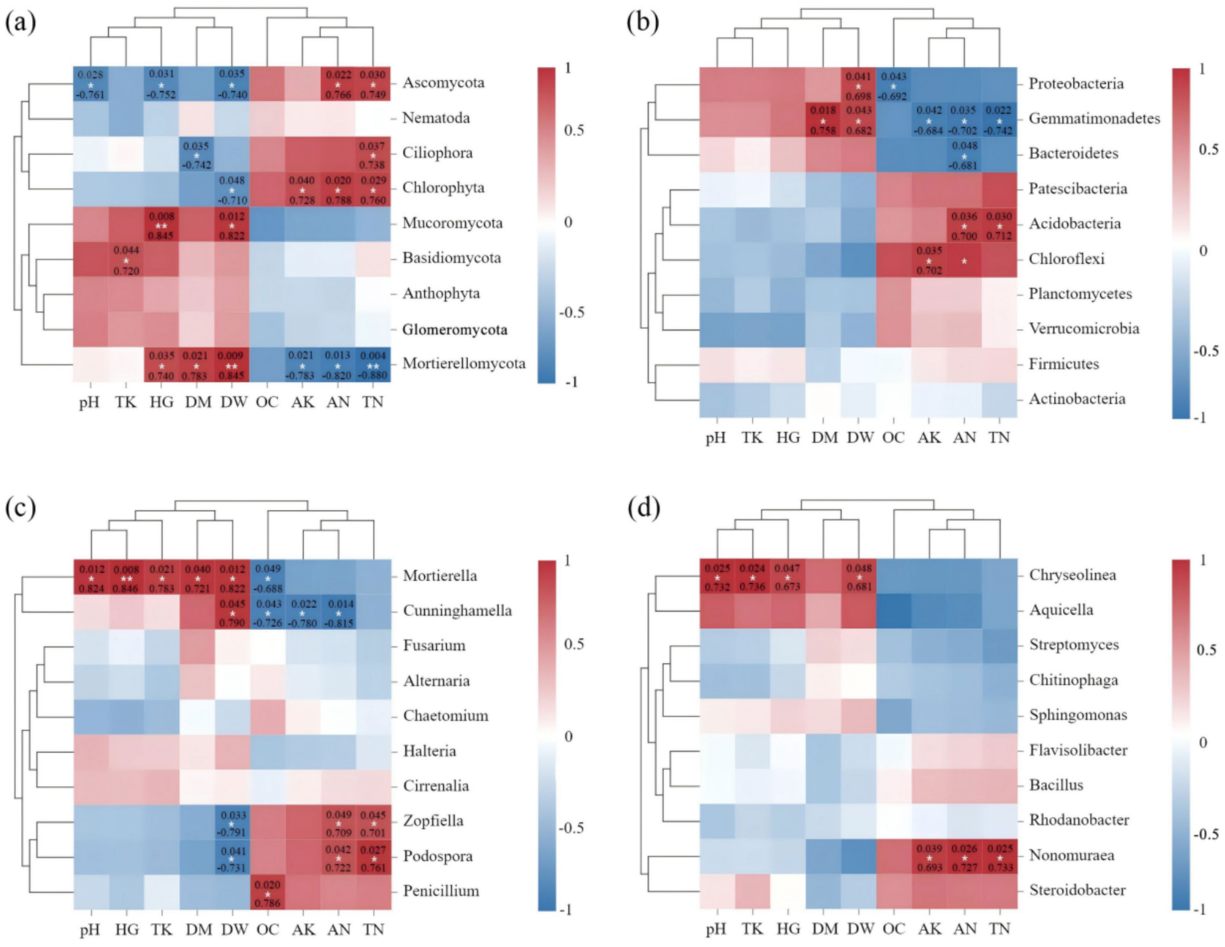
Heat map analysis of the correlation between the species composition of soil fungi **(A,C)** and bacteria **(B,D)** at the phylum **(A,B)** and genus **(C,D)** levels and soil nutrients and plant growth index. Here, OC, TN, AN, TK, AK HG, DM, and DW represent organic matter, total nitrogen, available nitrogen, total potassium, available potassium plant height, plant ground diameter, and dry weight, respectively. The legend on the right is the color interval for different *R* values. The number of asterisks indicates the degree of correlation (*p* < 0.05): *p ≤ 0.05, **0.05 < *p* ≤ 0.01, ***p ≤ 0.001.

Spearman correlation analysis further showed that fungi were more closely related to environmental factors than bacteria in the rhizospheric soil of *C. cajan*. Specifically, *Ascomycota* that the main fungal phyla, was significantly negatively correlated with pH (*p* = 0.028, *R* = −0.761), HG (*p* = 0.031, *R* = −0.752), and DW (*p* = 0.035, *R* = −740), while was significantly positively correlated with AN (*p* = 0.022, *R* = 0.766) and TN (*p* = 0.030, *R* = 0.749), respectively (Figure 6A). *Mortierellomycota* and *Mucoromycota* were significantly positively correlated with plant growth indexes, while *Mortierellomycota* was significantly negatively correlated with AK (*p* = 00.021, *R* = −783), AN (*p* = 0.013, *R* = −820) and TN (*p* = 0.004, *R* = −0.880), respectively (Figure 6A). At the genus level (Figure 6C), *Mortierella* was significantly positively correlated with plant growth indexes (*p* < 0.05) and pH (*p* = 0.012, *R* = 0.824), whereas *Mortierella* (*p* = 0.049, *R* = −0.688) and *Penicillium* (*p* = 0.020, *R* = 0.786) were significantly correlated with OC. Besides, soil pathogenic fungi, such as *Zopfiella* and *Podospora*, were significantly positively correlated with TN and AN, while significantly negatively correlated with DW. In the bacterial phylum community (Figures 6B,D), *Proteobacteria* was significantly negatively correlated with OC (*p* = 0.043, *R* = −0.692), while was significantly positively correlated with DW (*p* = 0.041, *R* = 0.698). Besides, *Acidobacteria* was significantly positively correlated with AN (*p* = 0.036, *R* = 0.700) and TN (*p* = 0.030, *R* = 0.712), respectively. At the genus level (Figure 6D), *Steroidobacter* belong to *Proteobacteria*, was positively correlated with pH, TK, HG, and DW. *Nonomuraea* was significantly positively correlated with AK (*p* = 0.039, *R* = 0.693), TN (*p* = 0.026, *R* = 0.727), and AN (*p* = 0.025, *R* = 0.733). *Bacillus* was not significantly correlated with soil environmental variables.

### 3.6 Prediction of fungal (FunGuild) and bacterial (FAPROTAX) ecological functions

**Figure 7:**
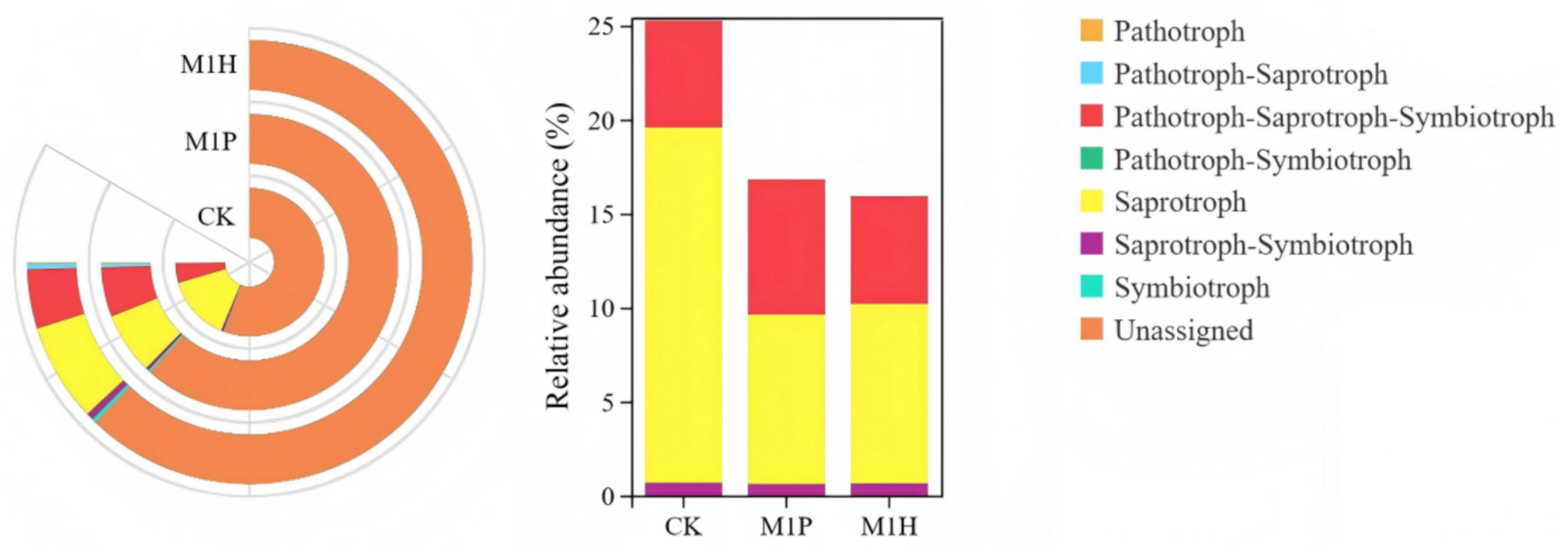
Functional differences of soil fungal communities with different microbial inoculants at trophic level.

**Figure 8:**
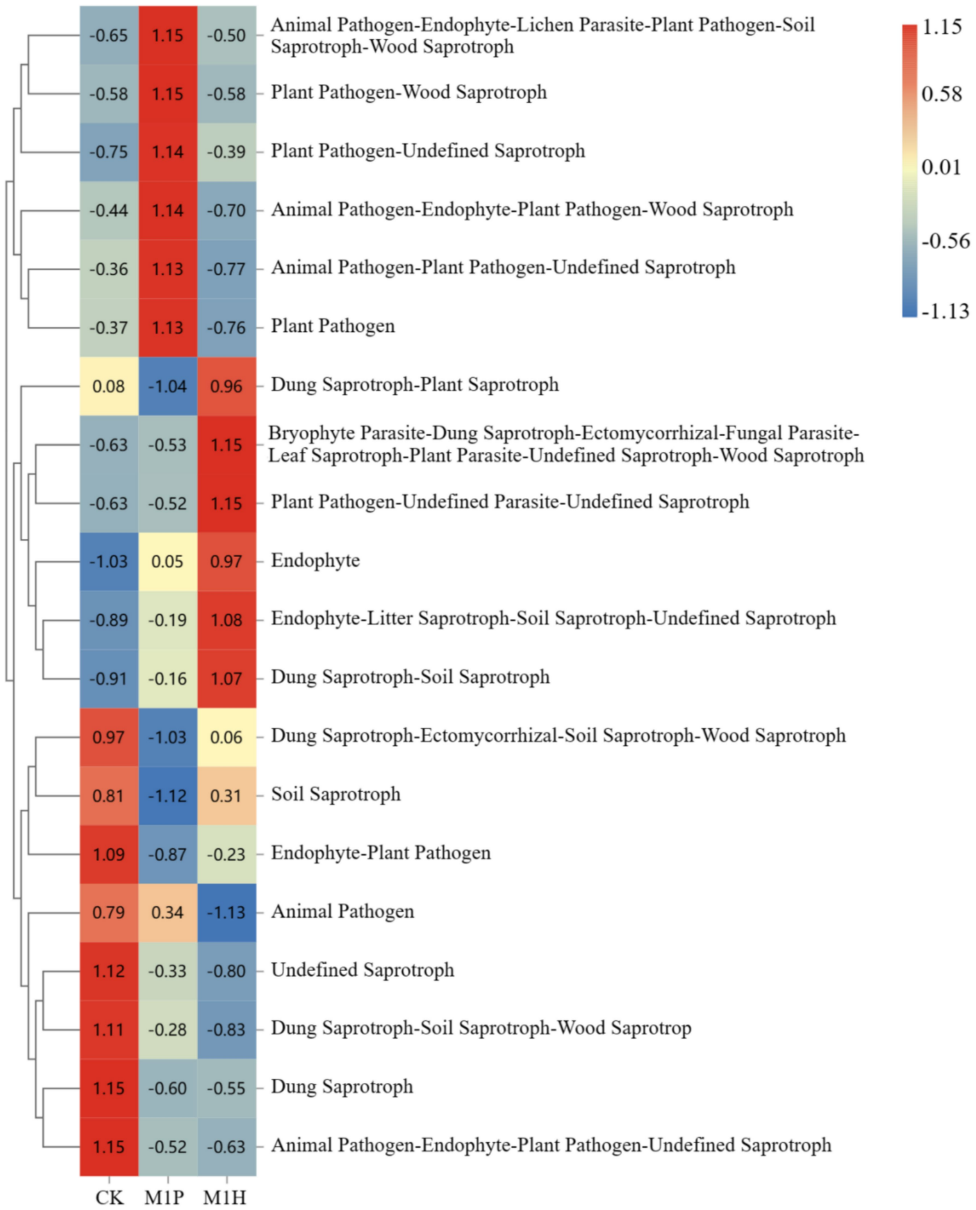
Analysis of community function heat map based on FunGuild at guild level. The legend on the right is the color interval for different R values. The number in the square indicates the specific *R* values.

The functional abundance of fungal communities of *C. cajan* rhizospheric soil from two treatments and the CK group were identified using ITS rDNA gene amplicon data and FunGuild (Figure 7). At the trophic level, the functional types of soil fungal communities could be divided into seven categories, including Saprotroph, Pathotroph-Saprotroph-Symbiotroph, Saprotroph-Symbiotroph, Symbiotroph, Pathotroph, Pathotroph-Saprotroph, and Pathotroph-Symbiotroph. Compared with CK, M1P and M1H treatments significantly increased the function of Unassigned, increasing by 8.23 and 8.89%, respectively. Meanwhile, Saprotroph, emerged as the most dominant soil fungal community, was significantly decreased by 9.89 and 9.35% under M1P and M1H treatment, respectively. Besides, Pathtroph-saprotrophs-symbiote, emerged as the second most dominant function of the soil fungal community, increasing its abundance under M1P and M1H treatments. The other functional abundance of fungal communities was less than 1%. At the guild level (Figure 8), the functional abundance of undefined Saprotroph, Dung Saprotroph, and Dung Saprotroph-Plant Saprotroph in saprotroph altered significantly. Compared with CK, M1P and M1H treatments decreased the Undefined Saprotroph by 2.01% (*R* = −0.33) and 2.67% (*R* = −0.80), respectively. Meanwhile, Dung Saprotroph abundance decreased by 7.05% (*R* = −0.60) and 6.87% (*R* = −0.55). The M1P treatment significantly decreased Dung Saprotroph-Plant Saprotroph by 0.54% (*R* = −1.04), while M1H treatment significantly increased it by 0.42% (*R* = 0.96), which could be one of the reasons for the difference in the M1P and M1H treatments. Moreover, M1P and M1H treatments increased the functional abundance of Animal Pathogen-Endophyte-Lichen Parasite-Plant Pathogen-Soil Saprotroph-Wood Saprotroph in Pathotroph-Saprotroph-Symbiotroph by 1.59% (*R* = 1.15) and 0.13% (*R* = −0.50). Meanwhile, the functional abundance of Animal Pathogen-Endophyte-Plant Pathogen-Undefined Saprotroph decreased by 1.32 and 1.40%, respectively under M1P and M1H treatments.

**Figure 9:**
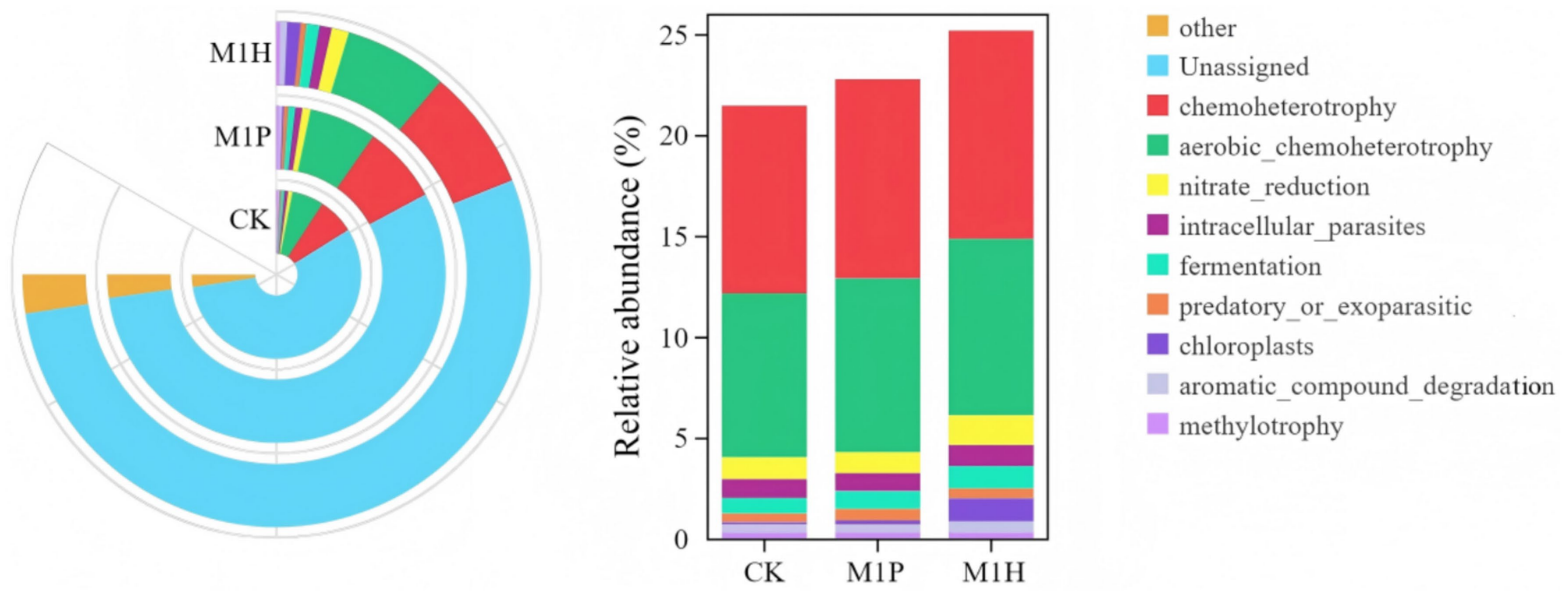
Functional differences of soil bacterial communities with different microbial inoculants.

**Figure 10:**
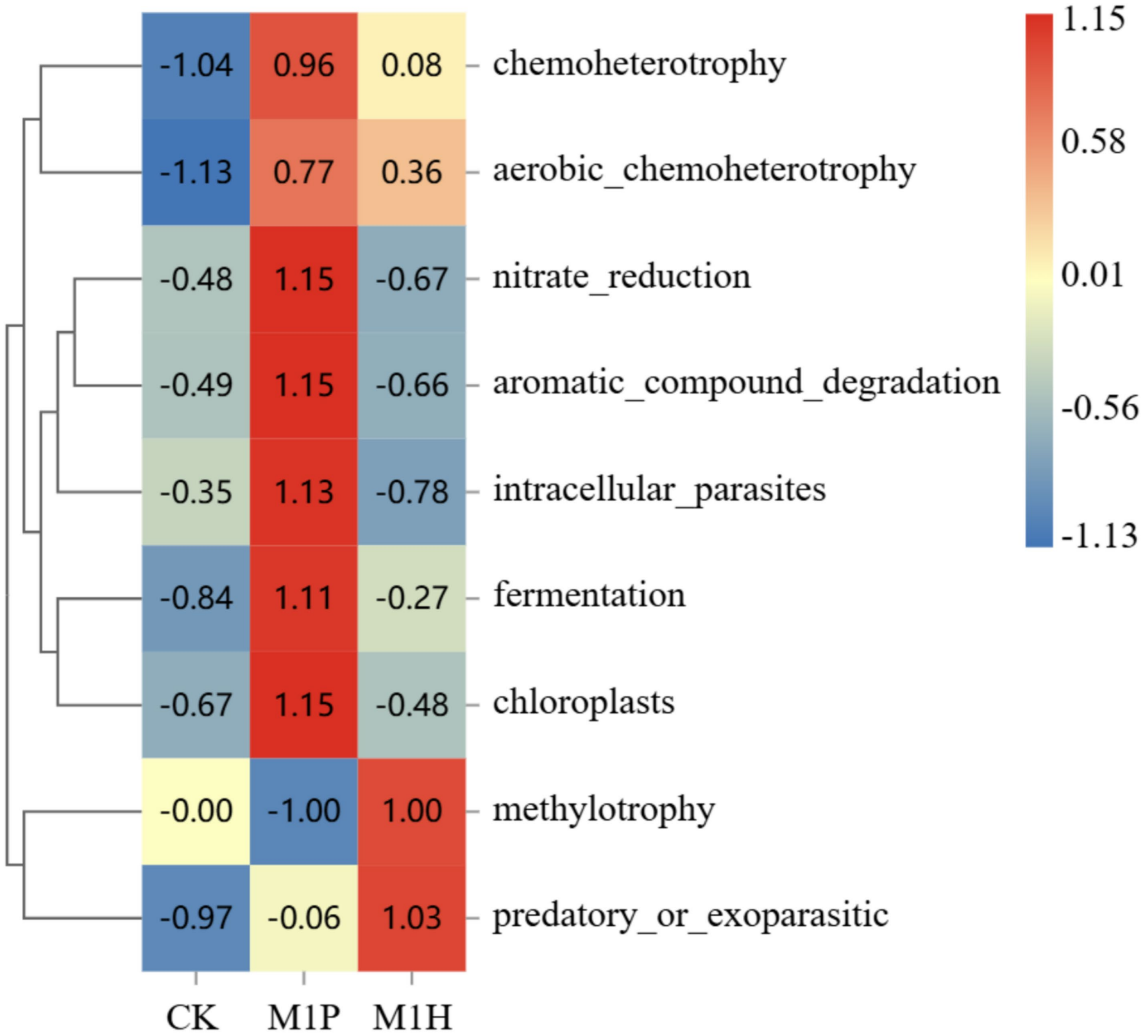
Analysis of community function heat map based on FAPROTAX. The legend on the right is the color interval for different *R* values. The number in the square indicates the specific *R* values.

The FAPROTAX functional prediction revealed differences in microbial functions between the CK treatment and the two inoculant treatments (M1P and M1H) (Figures 9, 10). The functional types of soil bacterial communities were categorized into seven categories, including chemoheterotrophy (9.30% ~ 10.32%), aerobic_chemoheterotrophy (8.14% ~ 8.75%), nitrate_reduction (1.03% ~ 1.46%), intracellular_parasites (0.89% ~ 1.04%), fermentation (0.78% ~ 1.11%), predatory_or_exoparasitic (0.42% ~ 0.57%), chloroplasts (0.11% ~ 1.14%), aromatic_compound_degradation (0.39% ~ 0.57%), and methylotrophy (0.33% ~ 0.34%) (Figure 9). Among these, chemoheterotrophy and aerobic_chemoheterotrophy were identified as the most dominant types of bacterial functions across all treatments. Notably, the abundance of chemoheterotrophy and aerobic_chemoheterotrophy was higher in the M1P and M1H treatments compared to the CK. In contrast, the abundance of the remaining five functions of bacterial community was less than 2% across all treatments.

Functional thermogram of analysis of the bacterial community (Figure 10) showed that M1P treatment showed enrichment in nitrate_reduction, aromatic_compound_degradation, intracellular_parasites, fermentation, and chloroplasts (*R* > 1.00). M1H treatment was enriched in methylotrophy and predatory_or_exoparasitic (*R* > 1.00). These results indicated that native microbial inoculation significantly affects the functional profile of the bacterial community in the rhizosphere soil of *C. cajan*. The enhanced microbial functions in the M1P and M1H treatments highlight the potential of these inoculants to modulate soil microbial activity effectively.

## 4 Discussion

### 4.1 Effect of M1H applied on soil properties

Soil nutrients, a basic index to evaluate soil fertility, could be used to determine the overall level of soil quality, which affects the structure of the soil microbial community, and finally affects the plant’s growth (Shao et al., 2020; Yin et al., 2021). Some reports indicated that microbial inoculants containing beneficial microbes can directly or indirectly modify soil physicochemical properties (Wei et al., 2018; Zhuang et al., 2021). For instance, soil pH is one of the most critical environmental factors affecting soil quality (Zheng et al., 2022). Previous studies shown that *Bacillus*-containing microbial inoculants significantly alleviated the alkalization of strawberry rhizosphere soil, increased the biomass, and improved fruit quality (Li et al., 2022). Similarly, in the current study, the native *S. marcescens* (M1P) and the native mixed microbe inoculants (M1H) alleviated soil acidification, and increased soil pH, in line with previous findings (Zheng et al., 2022). This effect could be attributed to decreased availability of available nutrients, such as AK, and AN likely caused by the activity of proton-coupled solute transporters and the anion uptake, which increase soil acidification (Hinsinger and Jaillard, 2010; Wong et al., 2008). Moreover, the application of microbial inoculants increase the consumption of soil carbon by microbial communities and plants, improve microbial metabolic activities, and regulate the stability of soil pH (Yin et al., 2021). Previous studies suggests that the application of organic inoculants and microbial inoculants improve the plant absorption of soil nutrients, decrease the contents of available nutrients in soil, (e.g., AK, AN, and AP), and thereby promote plant growth (Li Q. et al., 2019; Li Y. et al., 2019; Virk et al., 2022). In this study, we observed that M1H treatment significantly decreased AK and AN contents in the soil by 40.31 and 18.84%, respectively, and promoting the growth of *C. cajan* (plant height, plant ground diameter and dry weight). This reduction in available nutrients may result from microbial inoculants altering the soil microbial community structure, enhancing the microbial metabolic process, and increasing nutrient uptake by plants, consistent with previous findings (Li et al., 2022; Soong et al., 2018). Furthermore, Soussana and Lemaire (2014) and Wang C. et al. (2016) reported that microbial inoculants intake increased the underground carbon distribution in plants and soil carbon absorption, decreasing the OC content. Consistent with these studies. M1H treatment in this study significantly decreased the content of OC by 21.89% compared to CK, indicating increased the consumption and distribution of underground carbon sources by *C. cajan*, in line with previous findings (Li et al., 2022; Soussana and Lemaire, 2014; Wang C. et al., 2016). Interestingly, while the M1H treatment decreased TN and TK contents, potentially affecting AN and AK levels, the M1P treatment showed lower AN and AK contents than the CK treatment. The OC content in the soil showed a similar trend with AN and AK under M1H treatment. In addition, She et al. (2017) and Yin et al. (2013) reported that *Bacillus* can transform insoluble nutrients into effective nutrients needed by plant growth, and improve nutrient availability in the soil. However, the application of *Bacillus* also increases nutrient consumption of by microbes and plants, reflecting the true nutrient dynamics in the soil. These findings highlight the importance of native mixed microbial inoculants (M1H) in increasing the growth of *C. cajan* and provide insights into the mechanisms by which M1H affects soil nutrient dynamics. This study highlights the potential of microbial inoculants in sustainable agricultural practices, particularly for plant development.

Indeed, exogenous microbial inoculants have been employed to correlate plants growth to soil nutrients (Li et al., 2022; Zhuang et al., 2021). However, in the current research, most annotations focus on the promotion effect of soil nutrient enrichment on plant growth, but ignore the research on the balance relationship between plant absorption and soil eutrophication by exogenous strains. Moreover, *C. cajan*, as pioneer tree species for the prevention and control of soil erosion, but has received relatively less research attention (Lazcano et al., 2021). In our study, the relationship between the decrease of soil nutrients in *C. cajan* rhizosphere after M1H treatment and the benefit of plant growth confirmed the improvement of *Cajanus cajanus*’ ability to absorb soil nutrients to some extent. Additionally, due to the relative dearth of relevant data, and considering that the internal relationship between endogenous microbes and nutrient absorption of *C. cajan* has not been involved in the current research, this limitation hinders the current research, especially the changes of nutrients in *Cajanus cajanus* plants. Therefore, it is necessary to explore the changes of nutrients in *C. cajan* under M1H treatment to confirm the viewpoint and provide scientific protection for this study.

### 4.2 Effect of M1H on fungal and bacterial communities’ structure and function

Soil microbes are a vital part of the soil ecosystem, playing crucial roles in promoting plant growth and maintaining the micro-ecological environment of the soil (Liu et al., 2018; Luo et al., 2017; Song et al., 2022). In this study, it was observed that M1P and M1H treatments showed different trends in soil diversity indexes. Some previous studies showed that the application of microbial inoculants stimulated or induced the development of rhizosphere soil microbes, and thereby enriched the richness and diversity of soil microbial communities (Li et al., 2022; Zhuang et al., 2021), while others reported that although the application of microbial inoculants promoted plant growth and crop yield, it may simultaneously lead to a reduction in the diversity and richness of soil microbes. This reduction occurs because exogenous microbial inoculants, as an aggregate of the same microbe, intensifying competition among soil microbes within a short time, making the microbes with weak adaptability unable to cope with environmental changes, ultimately resulting in the reduction of soil richness and diversity (Cheng et al., 2021; Yao et al., 2003). These findings highlight that the variable and multi-directional effects of exogenous microbial inoculants on soil microbial communities. Understanding the potential functions of native microbial inoculants, particularly their effect on rhizosphere microbial diversity, offers a pathway to optimizing plant growth.

We then sought to understand the mechanism behind the ability of the rhizosphere microbiome to promoting the growth ability of *C. cajan* under inoculation with M1P and M1H inoculants. In this study, we observed that different microbial inoculants did alter the soil fungal community structure. *Ascomycota*, *Mucoromycota*, *Ciliophora*, *Mortierellomycota*, and *Chytridiomycota* were still the dominant phyla in the soil fungal community, and their abundances changed significantly, in line with previous findings (Wang H. et al., 2016; Zhou et al., 2015). Notably, *Mucoromycota* instead of *Ascomycota* emerged as the most abundant fungal phylum under M1H treatment, whereas the M1P treatment did not show this trend. This due to showed that *Ascomycota* and *Mucoromycota* could be significantly greatly influenced by the level of soil nutrients, mainly by OC, TN and AN, and *Mucoromycota* was negatively correlated with AN (*p* = 0.013, *R* = −0.820) and TN (*p* = 0.004, *R* = −0.880), respectively, and was positively correlated with plant growth indexes by using Spearman analysis (Figure 6A). Besides, some studies showed that the affects of *Mucoromycota* not always depend on high nutrient levels in the soil environment, but also on the nutrient content of the background soil (Nie et al., 2018). Nguyen et al. (2016) further reported that the abundance of *Mucoromycota* in soil was significantly different under different N levels and high N levels significantly restrained the abundance of *Mucoromycot* (Nguyen et al., 2016; Nie et al., 2018). In this study, the results showed that M1H treatment significantly decreased *Ascomycota* abundance in the soil, and the soil nutrient contents, including TN, TK, AN, and AK, corroborating previous findings (Nguyen et al., 2016). Furthermore, FunGuild analysis indicated that the application of M1P and M1H microbial inoculants significantly decreased the Saprotrophs abundance in the soil fungal community. This reduction was likely due to decreased *Ascomycot*a abundance in the soil resulting from the decreased level of soil nutrients after the addition of microbial inoculants, as *Ascomycota* contains a large number of microbial communities involved in saprophytic or symbiotic functions (Abbasi et al., 2021; Cheng et al., 2021). The reduction in soil nutrients following microbial inoculant application contributed to this shift in fungal community structure. These results suggest that applying native microbial inoculants, coupled with careful management of soil N levels, promotes the transformation of dominant soil microorganisms, improving the growth of *C. cajan*.

According to previous reports, beneficial microbial inoculants can optimize soil community structure, improve the soil micro-environment, and promote plant growth (Kaur and Garg, 2020; Li et al., 2022). For instance, the application of microbial inoculants containing *Penicillium* have been shown to significantly increase the abundance of *Mortierella*, thereby promote the growth of *I. matsum* (Zhuang et al., 2021). Similarly, *Bacillus*-containing inoculants can increase the abundance of beneficial microbes, such as *Firmicutes*, *Chloroflexi*, *Bacillus,* and *Actinomadura*, while inhibiting the abundance of pathogenic fungi, like *Fusarium* and *Phytophthora* in soil, and increasing strawberry yield (Li et al., 2022). While these studies highlight the contributions of microbial inoculants to soil community structure and the soil microbial environment, research on compound microbial inoculants combining *S. marcescens* and *P. polymyxa* remains limited. In this study, we observed that the application of M1H significantly increased the abundance of beneficial microbes, particularly *Cunninghamella* in the soil, which was positively correlated with the growth of *C. cajan*. The increased abundance of *Cunninghamella* likely contributed to enhanced nutrient consumption by *C. cajan*. Moreover, foreign microbial inoculants are known to induce changes in the structure of the soil microbial community structure. In this study, the most significant changes in the fungal community were the a decrease in the relative abundance of *Ascomycota* and an increase in *Mortierellomycota*, with M1H treatment significantly altered these relative abundances of *Ascomycota* and *Mortierellomycota* by 32.00 and 42.16%. Previous studies have indicated that *Ascomycota* is often more abundant in diseased soils, whereas *Mortierellomycota* is associated with healthy soils, and the relative abundance of *Mortierellomycota* was confirmed to have a significant relationship with *Penicillium* (Yuan et al., 2020; Zhuang et al., 2021). The genetic characteristics of *Mortierella* highlight its ability to degrade toxic organic matter, contributing to soil health (Zhuang et al., 2021); Furthermore, *Mortierella* has been shown to increase the level of indole acetic acid levels in plants, thereby enhancing plant biomass. These findings strongly support the role of *Mortierella* as a beneficial microorganism that promotes soil improvement and plant growth. The observed increase in *Mortierella* abundance following M1H application represents a positive shift in the soil microbial community, further validating the effectiveness of the M1H microbial inoculant in improving soil health and supporting the growth of *C. cajan*.

In this study, we further investigated the effects of native mixed microbial inoculants on potential microbial communities, as they may represent an important factor affecting the growth of *C. cajan*. The abundance of *Zopfiella* and *Podospora* (both belong to *Ascomycota*) were significantly decreased under M1H treatment, and were negatively correlated with plant growth indexes indices based on Spearman analysis (Figure 6C). *Zopfiella* and *Podospora* belong to *Chaetomiaceae* and *Lasiosphaeriaceae*, respectively, and both belong to Ascomycota, widely exist in soil feces contain important pathogens or saprophytic fungi that can may negatively impact plant growth (Li Q. et al., 2019; Li Y. et al., 2019). Furthermore, Ma et al. (2024), by analyzing 82 animal feces samples from different sources, reported that *Zopfiella* and *Podospora* in wild budgerigar feces showed pathogenic characteristics, while pathogenic effects of *Zopfiella* and *Podospora* in feces from other sources were less apparent. These findings suggest that the M1H microbial inoculant can optimize the soil microenvironment by promoting, the abundance of beneficial microbes while suppressing the abundance of potentially microbes, aligning with previous studies (Kaur and Garg, 2020; Li et al., 2022). RDA revealed that *Zopfiella* and *Podospora* were positively correlated with TN and AN levels, indicating that high soil nitrogen levels could elevate the abundance of pathogenic microbes (Wei et al., 2018). Moreover, the soil pH value (*R* = 0.012, *p* = 0.824) and TK (*R* = 0.021, *p* = 0.783) were identified as key factors affecting the abundance of *Mortierella*. In this study, M1H treatment significantly decreased the abundance of genes associated with the saprotroph function of soil flora and the relative abundance of *Zopfiella* and *Podospora*, both members of *Ascomycota*. This reduction suggests that *Zopfiella* and *Podospora* may play a primary role in saprotrophic functions. However, the observed changes in the abundance of these fungi in the rhizosphere soil of *C. cajan* do not provide evidence regarding their potential to cause disease or the overall disease status of the soil. While the roles of *Zopfiella* and *Podospora* in the rhizosphere remain unclear, their correlation with plant growth indices suggests that they may act as potential factors affecting the growth of *C. cajan* (Figure 6C). Therefore, these fungi have been classified as potential soil microbes in this study. Despite the observed associations, a lack of targeted research limits the understanding of the pathogenicity and mechanisms of *Zopfiella* and *Podospora* in the context of *C. cajan* physiology. While this study establishes a foundation by highlighting the relationship between their abundance and plant growth, further research is needed to confirm their pathogenic roles and explore their mechanisms of action. The findings presented here offer a new perspective for future investigations into the potential pathogenicity of *Zopfiella* and *Podospora*. In summary, the application of M1H inoculants altered the relative abundance of beneficial and potentially harmful microbes in the soil, improving the soil microenvironment and explaining changes in soil nutrient factors. Compared to studies on single microbial inoculants, this research marks a significant advancement by demonstrating the broader impacts of mixed microbial inoculants on soil microbial communities and plant growth.

### 4.3 Internal factors affecting the growth-promoting ability of M1H

The application of microbial inoculants has been shown to affect the soil nutrients, alter the soil microbial community structures, and impact plant growth (Cheng et al., 2020; Li et al., 2022; Zhang et al., 2019). In this study, we observed that the addition of M1H inoculants significantly affected the microbial diversity and community structure of *C. cajan* rhizosphere soil, with a greater impact observed on soil fungi compared to bacteria. This suggests that fungi may play a key role in the analyzed soil environment in promoting the growth of *C. cajan*. At the genus level, the abundances of *Cunninghamella*, *Zopfiella*, and *Podospora* were significantly changed under the M1H treatment compared to CK. Notably, the abundance of *Cunninghamella* was positively associated with *C. cajan* growth, while *Zopfiella* and *Podospora* showed negative correlations. OC and AN were identified as the primary environmental factors affecting the abundance of these genera. Moreover, while no significant differences were observed in the abundances of *Zopfiella* and *Podospora* between M1H and M1P treatments, *Cunninghamella* abundance was significantly higher in the M1H treatment. This increase corresponded with enhanced growth of *C. cajan*, indicating that the superior growth-promoting effects of M1H compared to M1P may be attributed to the increased abundance of *Cunninghamella*. Differences were also observed at the bacterial genus level between M1H and M1P treatments, with genera such as *Chryseolinea*, *Nonomuraea,* and *Chitinophaga* showing significant variations. This may be related to the role of trace elements in promoting interactions between microbes (Fang et al., 2020; Toffa et al., 2021). Previous studies have demonstrated that host microbes usually exist in plants and the nutrient-rich rhizospheric soil environments increase the absorption of soil nutrients through roots. It is conducive to the decomposition of organic matter and the absorption of available nutrients, thereby promoting the growth and development of plants (Ingram et al., 2005; Li et al., 2022; Soong et al., 2018). These findings align with our study, which highlights the potential roles of *Cunninghamella*, *Zopfiella*, and *Podospora* in the growth of *C. cajan*. The results suggest that inoculating the soil micro-ecological environment with native microbial inoculants can reduce biological competition, optimize soil conditions, and promote plant growth. This highlights the importance of localized microbial inoculation strategies in improving plant performance and soil health.

## 5 Conclusion

Our research examined the impact on *C. cajan* growth, soil nutrients and soil rhizosphere microbiomes by pot experiment with different native microbial inoculants. The results showed that both M1P and M1H treatments significantly promoted the growth of *C. cajan*, but M1H treatment showed a higher growth-promoting ability than M1P treatment, and increased the consumption capacity of soil OC, AN, and AK contents. Meanwhile, M1H treatment led to substantial alterations in the soil microbial community that were more effective in promoting *C. cajan* growth, particularly through increasing beneficial genera (e.g., *Cunninghamella*, *Mortierella*, *Chryseolinea*, *Chrysolina*, and *Bacillus*) and decreasing potential genera (e.g., *Zopfiella* and *Podospora*). Among then, *Cunninghamella*, *Zopfiella*, and *Podospora* were the key bioindicators for influencing *C. cajan* growth by Spearman correlation analysis. The study also proved that OC, AK, AN, and TN were the key environmental factors influencing *Cunninghamella*, *Zopfiella*, and *Podospora*. Notably, the effect of M1H inoculation on soil fungal community was higher than that of bacterial community at the genus levels, which indicates that the change of soil fungal community after M1H inoculation was more active than that of bacteria. In summary, the native mixed microbial inoculants (M1H) enhanced the consumption of soil nutrients by *C. cajan*, purified the soil micro-environment, and thereby promoted the growth of *C. cajan*. This study confirmed the feasibility and effectiveness of mixed microbial inoculants in promoting plant growth, which provides a theoretical basis and sustainable management strategy for the cultivation of *C. cajan* and the development of the agriculture husbandry industry.

## Data Availability

The datasets presented in this study can be found in online repositories. The names of the repository/repositories and accession number(s) can be found in the article/Supplementary material.

## Footnotes

^1^ https://cran.r-project.org/

^2^ http://www.omicsmart.com
