# Supplementary material for: Native mixed microbe inoculants (M1H) optimize soil health to promote Cajanus cajan growth: the soil fungi are more sensitive than bacteria
Source: Front Microbiol. 2025 Mar 5;16:1521064. doi: 10.3389/fmicb.2025.1521064 (PMC11919897; doi:10.3389/fmicb.2025.1521064)
Supplement: Supplementary file 1 [file Data_Sheet_1.pdf]

Supplementary material

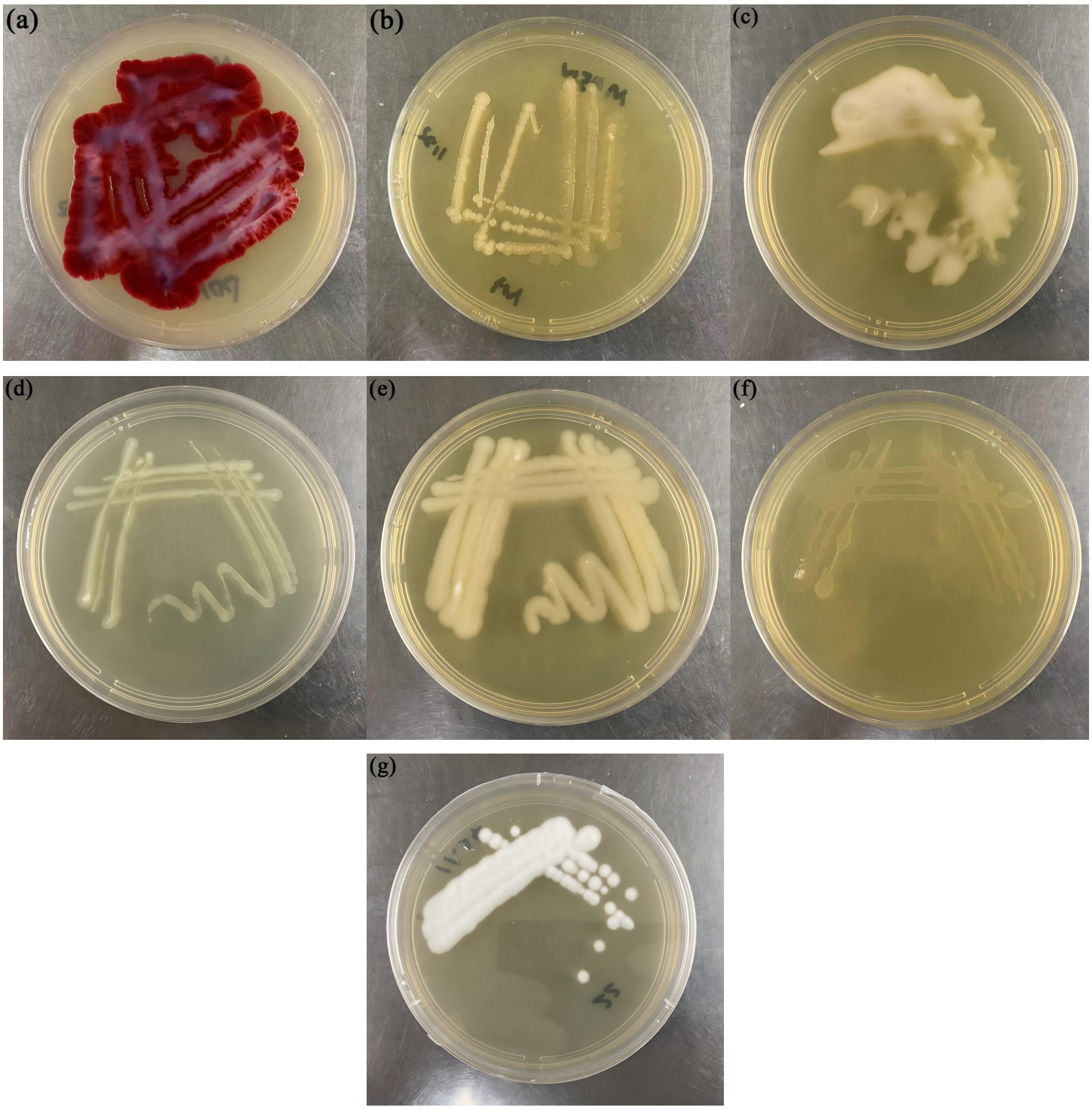

Fig. S1. M1P: *Serratia marcescens* (a), M45N: *Paenibacillus polymyxa* (b), M1N (c), M2N (d), M3N (e), M2P (f), and M45P (g). Here, only the strains used in this study (M1P and M1H) were identified.

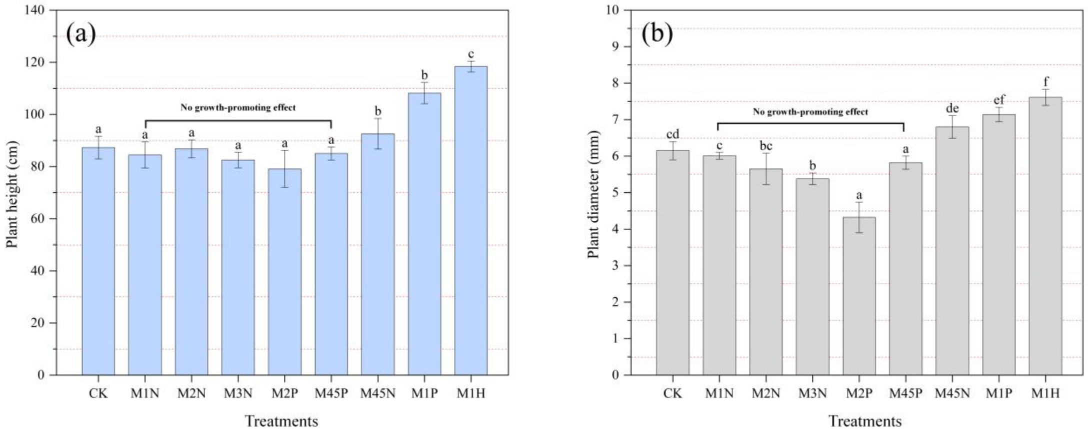

Fig. S2. Effect of application of different native microbial inoculants on the growth of *Cajanus cajan*.

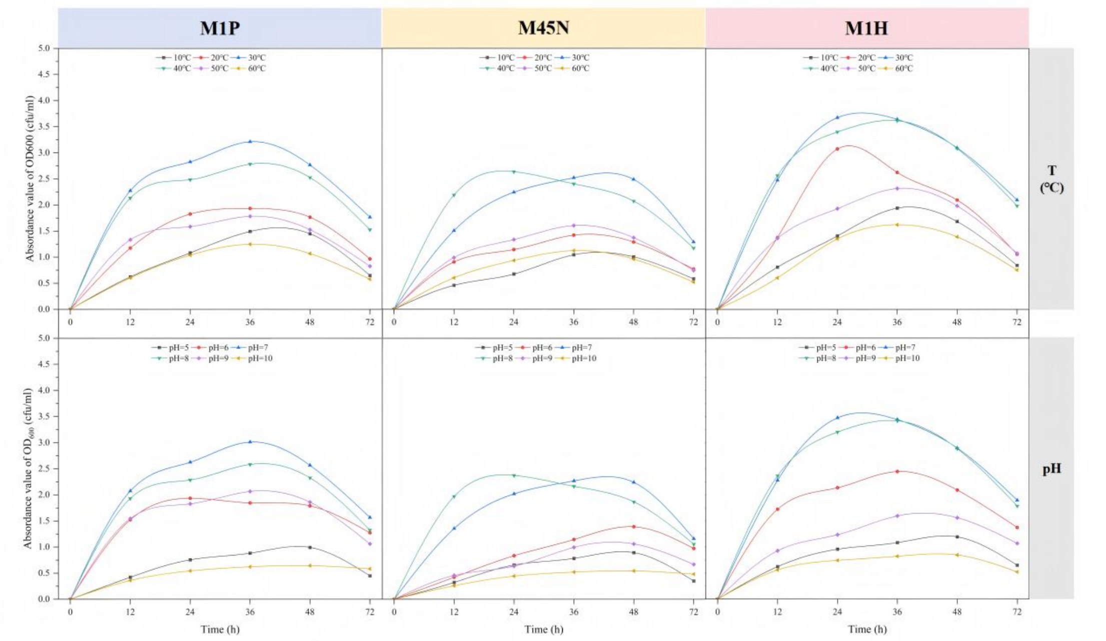

Fig. S3. Growth of different microbial inoculants strains under temperature and pH stress.

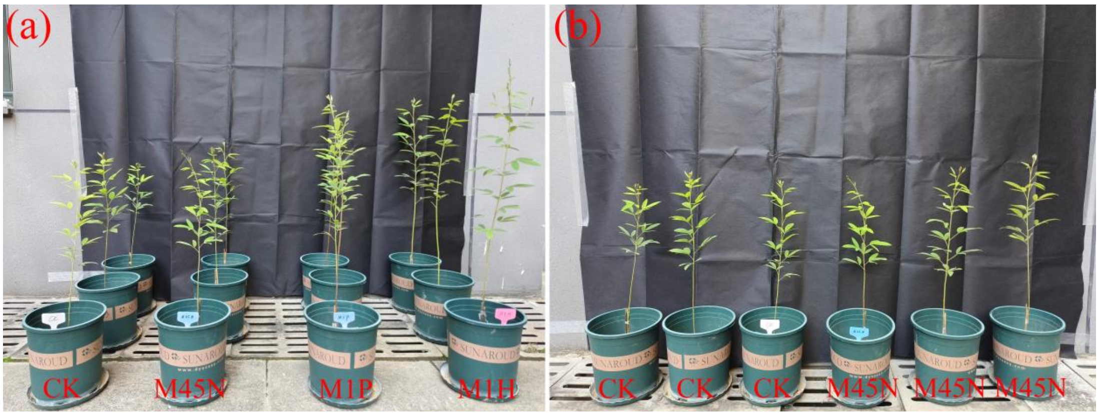

Fig. S4. Pot diagram of application of different native microbial inoculants (M1P, M45N, and M1H treatments) on the growth of *Cajanus cajan*.

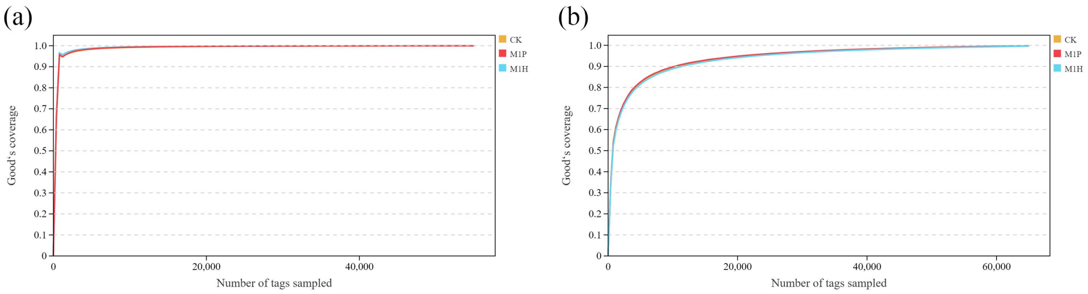

Fig. S5. Variation in the dilution curves of soil fungi (a)and bacteria (b) under different microbial inoculants.

Tab. S1. Soil physical and chemical properties of original values.

	pH	OC (g/kg)	TN (g/kg)	TK (g/kg)	AN (mg/kg)	AK (mg/kg)
Original values	6.72 ± 0.03	32.65 ± 1.56	3.20 ± 0.12	23.42 ± 0.42	246 ± 6.02	285.76 ±7.56
